# Multiple regression analysis of the craniofacial region of Chinese Han people using linear and angular measurements based on MRI

**DOI:** 10.1080/20961790.2016.1276120

**Published:** 2017-03-30

**Authors:** Chengzhi Li, Wei Wu, Bo Zhu, Xuefeng Liu, Ping Huang, Zhenyuan Wang, Ya Tuo, Fu Ren

**Affiliations:** a Health Science Center, School of Forensic Science and Medicine, Xi'an Jiaotong University, Xi'an, China; b Department of Anatomy, Institute of Biological Anthropology, Liaoning Medical University, Jinzhou, China; c Shanghai Key Laboratory of Forensic Science, Shanghai Forensic Service Platform, Institute of Forensic Science, Ministry of Justice, PRC, Shanghai, China; d Nuclear Medicine Department, The First Affiliated Hospital of Jiamusi University, Jiamusi, China; e Department of Biochemistry and Physiology, Shanghai University of Medicine and Health Sciences, Shanghai, China

**Keywords:** Forensic science, forensic anthropology, anthropometry, regression analysis, magnetic resonance imaging

## Abstract

The purpose of this study was to measure the craniofacial region of Chinese Han people in the linear and angular dimensions, and to analyse the effects on sex, age and body parameters (height and weight). All 250 individuals (86 males, 164 females) underwent a three-dimensional magnetic resonance imaging (MRI) scan, and the MRI data were imported into VG Studio MAX 2.2 software. Each linear and angular measurement in the craniofacial region was processed directly. Using SPSS 20.0 software, nine multiple regression equations were constructed, and all the adjusted *R*^2^ values were statistically significant (0.031–0.311). Multiple regression analysis showed that most craniofacial measurements of Chinese people were significantly correlated with height, weight or age. The multiple regression equations constructed will be helpful in anthropometric analysis and forensic inference.

## Introduction

With the development of new technology, many methods have been developed to analyse craniofacial morphology, such as high-resolution computed tomography (CT), three-dimensional laser scanning, digital imaging and geometry measurement analysis [1–3]. Magnetic resonance imaging (MRI) has been used widely as a diagnostic tool in clinical practice. Compared with X-ray and CT, the resolution of MRI is higher for soft tissues, and is free of radiation [4–6]. In recent years, it has also been used for measuring and analysing craniofacial morphology [7–11]. In some studies, craniofacial morphology has been associated with the factors of genes [12–14], sex [15–17] and hormone levels [18–20]. Other studies have determined that the surface area and volume of intracranial organs are closely related to age, some diseases, sex and race [1,21,22]. However, few studies have investigated the relationship of linear and angular measurements in the craniofacial region to height, weight, age and sex. Craniofacial measurement and analysis are significant factors in clinical diagnosis and forensic identification [23,24]. Therefore, the aim of this study was to measure the craniofacial region of Chinese people in linear and angular dimensions based on MRI and VG Studio MAX 2.2 software, and to analyse the linear correlations between cephalometric variables (linear and angular) and age, height and weight using SPSS 20.0 software. We also constructed multiple regression equations of the cephalometric variables (linear and angular) and analysed the differences in craniofacial measurements between males and females. The results of this study provide insight into craniofacial morphology and forensic inference.

## Materials and methods

### Subjects

From June to December 2012, 250 healthy volunteers were selected from adults of Han nationality including 86 males and 164 females (weight ranged from 147 to 192 cm; height ranged from 40 to 110 kg) in Liaoning, China. The age distribution of the volunteers is shown in Table 1. The inclusion criteria were: (1) aged 20–70 years; (2) being at least the third generation lived in China; (3) no congenital or acquired skull deformity. The exclusion criteria were: (1) metal in the body, such as a cardiac pacemaker, artificial valve or metal dentures; (2) history of claustrophobia or functional disorder of heat dissipation; (3) history of mental instability or sensitivity to noise. All participants provided written informed consent in accordance with the Human Research Committee of Liaoning Medical University.

**Table 1.** Age distribution of subjects.

Age group/year	Number
20–29	81
30–39	49
40–49	45
50–59	38
60–70	37

### Image acquisition

All subjects lay in a supine position, with their head in the Frankfort horizontal plane. MRI was performed at a field strength of 1.5 Tesla. The following steps were taken: T_1_W three-dimensional magnetization prepared rapid acquisition gradient echo sequences, T_1_WI 3D MPRAGE (TR = 2 000 ms, TE = 4.0–4.5 ms, TI = 1 100 ms, FOV = 256 mm × 256 mm, 192 sagittal 1.0 mm slices without gaps, flip angle [FA] 15, matrix size = 512 × 512 × 192, voxel size = 1 mm ×1 mm × 1 mm). The total scanning time was approximately 25 minutes.

### Image processing

**Figure 1.**
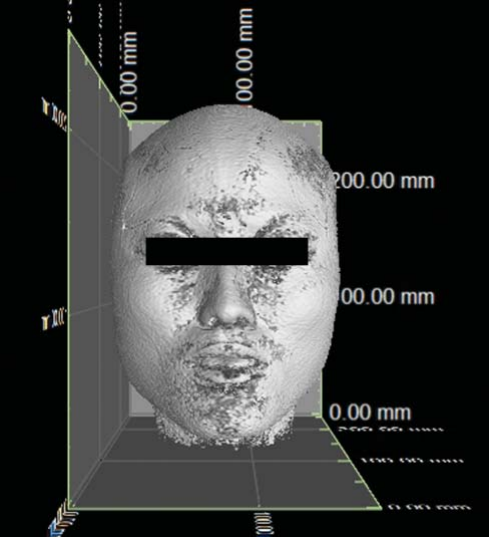
The stereogram of the three-dimensional reconstruction of MRI data.

**Figure 2.**
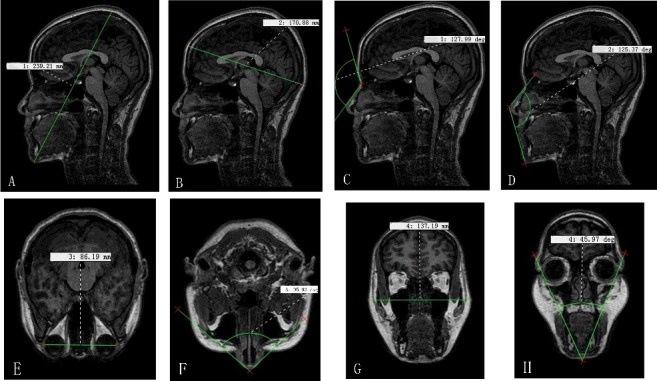
Linear and angular measurements were performed directly on the VG Studio MAX 2.2 software at three different dimensions. (A) Gn-V, (B) Tr-Op, (C) G-S-Prn, (D) S-Prn-Gn, (E) Ex-Ex, (F) Al-Prn-Al, (G) Ex-Ex, (H) Ex-Gn-Ex.

The MRI data of the 250 participants were imported into VG Studio MAX 2.2 software, and a three-dimensional reconstruction of the MRI data was built (Figure 1). To overcome variations, all the images were put in the same coordinate system. The picture's brightness and contrast were adjusted in a window with four partitions. Linear and angular measurements were taken with the measuring tool on the VG Studio MAX 2.2 software at three different dimensions (Figure 2). All measurements were recorded by one person. The anthropometric landmarks (Table 2) used for the measurement of each linear and angular dimension have been defined in previous studies [25–27]. The cephalometric variables used in this study are shown in Table 3.

**Table 2.** Symbols and definitions of anthropometric landmarks used.

Landmark	Symbol	Definition
Gnathion	Gn	The lowest point in the midline on the lower border of the chin
Vertex	V	The highest point on the head in the sagittal plane, with the head in the Frankfort horizontal plane
Trichion	Tr	Midpoint of the hairline
Opisthocranion	Op	The most prominent posterior point on the occiput
Exocanthion	Ex	The outer corner of the eye fissure where the eyelids meet
Zygion	Zy	The most lateral point on the zygomatic arch
Glabella	G	The cephalic surface point at the most prominent midline point between the eyebrows
Sellion	S	The deepest point located on the bottom of the nasofrontal angle
Alare	Al	The most lateral point on the nasal ala
Pronasale	Prn	The most protruded point of the nasal tip

**Table 3.** Cephalometric variables.

Variable	Description
**Linear (mm)**	
Gn-V	The longest linear distance between gnathion and vertex in the median sagittal plane
Tr-Op	The longest linear distance between trichion and opisthocranion in the median sagittal plane
Ex-Ex	The longest linear distance between the left and right exocanthion in the horizontal plane
Zy-Zy	The longest linear distance between zygion and zygion in the coronal plane
**Angular (°)**	
G-S-Prn	The angle between glabella-sellion and sellion-pronasale
S-Prn-Gn	The angle between sellion-pronasale and pronasale-gnathion
Al-Prn-Al	The angle between alare-pronasale and pronasale-alare
Ex-Gn-Ex	The angle between exocanthion-gnathion and gnathion- exocanthion in the coronal plane

### Reliability of measurements

Inter-rater reliability between individual raters was calculated using the intra-class correlation carried out on 10 brain scans before rating the images for the study. The ranges of these were as follows: Gn-V 0.90–0.98, Tr-Op 0.93–0.98, Ex-Ex 0.91–0.99, Zy-Zy 0.90–0.97, G-S-Prn 0.89–0.97, S-Prn-Gn 0.90–0.96, Al-Prn-Al 0.88–0.98, Ex-Gn-Ex 0.90–0.98. Intra-rater reliability was calculated using intra-class correlation on 10 scans rated at least 6 months apart and was in the range of 0.95–0.98 for all linear and angular measurements. The intra-class correlation was performed as follows: one-way model, single measure and consistency.

### Statistical analysis

Descriptive statistics including the mean and standard deviation (SD) were calculated for males and females separately. Significant differences between the sexes were assessed using Student's *t–*test, and probabilities of less than 0.05 were accepted as significant. Multiple regression analysis was carried out to investigate the relationship between the cephalometric variables and height, weight and age using SPSS 20.0 software.

## Results

**Figure 3.**
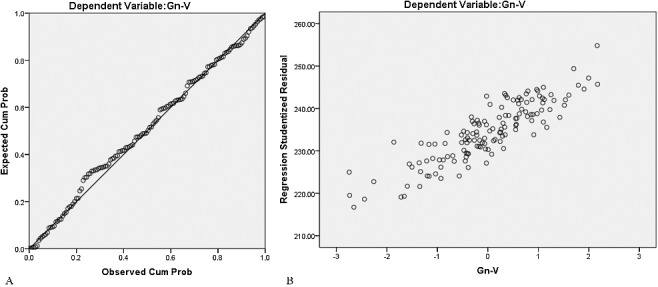
Normal probability plot A show the dependent variable (Gn-V) is in normal distribution, and scatter plot B shows the dependent variable (Gn-V) has equal variance.

The mean and SD of linear and angular anthropometric measurements for Chinese adults of Han nationality are shown in Table 4. There were significant differences in all the anthropometric measurements between the sexes except for Al-Prn-Al. Age and body parameters (height and weight) also had an effect on the craniofacial measurements. The results of the multiple regression analysis, with corresponding *r*, adjusted *R*^2^ and standard error (SE) of the estimate, are shown in Tables 5 and 6. All the dependent variables exhibited a normal distribution with equal variance. As an example, the normal distribution and equal variance of Gn-V are shown in Figure 3. In the multiple linear regression equations, height, weight and age served as independent variables, and the cephalometric variables served as dependent variables. The results show that Gn-V was significantly correlated with height and weight in both males and females; Tr-Op was significantly correlated with weight in males and age in females; Ex-Ex was significantly correlated with age in males and weight and age in females; Zy-Zy was significantly correlated with weight in both males and females; and G-S-Prn was significantly correlated with weight in females. The multiple correlation coefficients ranged from 0.192 to 0.583, the adjusted *R*^2^ ranged from 0.031 to 0.311, and the SE of the estimate ranged from 4.074 to 7.727. There were statistically significant differences in all the independent coefficients (*P* < 0.05).

**Table 4.** Descriptive statistics of means, standard deviations (SD) and Student's *t-*test of the differences between the sexes for eight cephalometric variables.

Cephalometric variables	Male (*n* = 86)	Female (*n* = 164)	*t*-Value	*P*-Value
Gn-V (mm)	246.62 ± 5.66	234.41 ± 7.14	10.74	<0.001
Tr-Op (mm)	178.07 ± 7.94** **	170.52 ± 7.24	7.48	<0.001
G-S-Prn (°)	130.36 ± 8.13	137.61 ± 7.37	−7.13	<0.001
S-Prn-Gn (°)	117.92 ± 5.49	120.32 ± 5.97	−2.51	<0.05
Ex-Ex (mm)	91.60 ± 4.29	88.59 ± 4.20	5.21	<0.001
Al-Prn-Al (°)	91.10 ± 5.16	90.15 ± 7.36	1.03	>0.05
Zy-Zy (mm)	131.67 ± 7.60	125.08 ± 7.53	6.25	<0.001
Ex-Gn-Ex (°)	42.01 ± 3.24	44.57 ± 3.23	−5.03	<0.001

**Table 5.** Multiple linear regression equations with corresponding *r*, adjusted *R*^2^ and standard error (SE) of the estimate in males.

Regression equations	*r*	Adjusted *R*^2^	SE
*Y*_1_ = 164.639 + 0.388*X*_1_ + 0.195*X*_2_	0.583	0.311	4.696
*Y*_2_ = 163.575 + 0.206*X*_2_	0.255	0.053	7.727
*Y*_3_ = 94.392 − 0.095*X*_3_	0.265	0.058	4.165
*Y*_4_ = 99.636 + 0.456*X*_2_	0.543	0.286	6.420

*Y*_1_ = Gn-V; *Y*_2_ = Tr-Op; *Y*_3_ = Ex-Ex; *Y*_4_ = Zy-Zy; *X*_1_ = Height; *X*_2_ = Weight; *X*_3_ = Age.

**Table 6.** Multiple linear regression equations with corresponding *r*, adjusted *R*^2^ and standard error (SE) of the estimate in females.

Regression equations	*r*	Adjusted *R*^2^	SE
*Y*_1_ = 147.400 + 0.467*X*_1_ + 0.219*X*_2_	0.484	0.223	6.187
*Y*_2_ = 166.930 + 0.131*X*_3_	0.192	0.031	7.125
*Y*_3_ = 84.289 + 0.134*X*_2_ − 0.107*X*_3_	0.279	0.066	4.074
*Y*_4_ = 105.665 + 0.358*X*_2_	0.362	0.125	7.051
*Y*_5_ = 149.528 − 0.220*X*_2_	0.229	0.046	7.217

*Y*_1_ = Gn-V; *Y*_2_ = Tr-Op; *Y*_3_ = Ex-Ex; *Y*_4_ = Zy-Zy; *Y*_5_ = G-S-Prn; *X*_1_ = Height; *X*_2_ = Weight; *X*_3_ = Age.

## Discussion

In earlier studies, cephalometric variables were measured by right-angle gauge, tapeline and other older-style tools [28]. With the development of imaging science, X-rays and CT are now used for craniofacial measurements. In recent years, MRI has also been used widely for measuring and analysing craniofacial morphology [7–11]. Compared with other measuring tools, MRI has unique advantages for craniofacial measurements. It is more flexible and reliable than other tools, and can be operated without radiation. MRI also offers higher resolution images of soft tissues [4–6]. In this study, using MRI and relevant software, we carried out quantitative analysis of craniofacial morphology to reveal further details of craniofacial features and variations. The multiple linear regression equations constructed in this study have great significance for craniofacial restoration, plastic surgery, and forensic inference.

Except for the mean value of Al-Prn-Al, all the measurements in this study were significantly correlated with sex (*P* < 0.05). The mean values of G-S-Prn, S-Prn-Gn and Ex-Gn-Ex were greater in females than in males. However, the four linear measurements were significantly greater in males than in females. This indicates craniofacial morphology is closely related to sex, which is in accordance with previous studies [15–17]. Using SPSS 20.0 software, nine linear regression equations were constructed, which showed that all measurements except Al-Prn-Al and Ex-Gn-Ex were significantly correlated with age or body parameters, such as height and weight. The angular measurement (G-S-Prn) was negatively correlated with weight in females. In contrast, the four linear measurements were on the whole positively correlated with height, weight or age in both males and females. Therefore, to some extent, the linear and angular measurements in the craniofacial region can reflect the age, height and weight of an individual. This may be helpful in the inference of forensic anthropology. The third multiple regression equation in Tables 5 and 6 shows that the average value of Ex-Ex has a significant negative correlation with age. This suggests that the value of Ex-Ex becomes smaller as the contractility of the extraocular muscles gradually weakens with age and the eyeballs move closer together. The fibrous protein of the eyeball also becomes indurated with increasing age; thus, the volume of the eyeball gradually decreases. All these factors may contribute to the decrease in the value of Ex-Ex as people become older.

Most previous studies [14–17] of craniofacial morphology have used qualitative analysis. In this study, we have used indicators not only to discuss the differences between males and females, but also to construct multiple linear regression equations to show the relationship between cephalometric variables and age, height and weight with quantitative analysis. One study [29] reported that in males in Yunnan, China, the average head length was 175.9 mm, head breadth was 141.1 mm, and total head height was 137.9 mm; but in females in Guizhou, China, the average head length was 179.2 mm, head breadth was 129.0 mm, and total head height was 128.6 mm. These results and the results of our study are mostly in accordance with the fact that males have a greater head length, head breadth and head height than females. However, the head length for females in Guizhou, China, was greater than for males in Yunnan, China. There are several possible reasons for this difference. First, it may be the result of different nationalities of the subjects. Second, the measurements were performed on the bones of dead bodies, and did not include soft tissue. Finally, it may be related to genetic factors. The craniofacial measurements of Chinese people may provide some data for developing a standard guide to appearance among Chinese people, which may be of significance in cosmetic surgery. The study of human morphology will develop further with the rapid development of new techniques and methods from the fields of genetics and computer applications.

## Conclusion

We found significant differences between the sexes in all anthropometric measurements, except for Al-Prn-Al, in Chinese Han people. Age and body parameters, such as height and weight, also had an effect on the craniofacial measurements. The multiple regression equations constructed will be useful for anthropometric analysis and forensic inference.
